# The transcriptional coactivator and histone acetyltransferase CBP regulates neural precursor cell development and migration

**DOI:** 10.1186/s40478-019-0849-5

**Published:** 2019-12-05

**Authors:** Melanie Schoof, Michael Launspach, Dörthe Holdhof, Lynhda Nguyen, Verena Engel, Severin Filser, Finn Peters, Jana Immenschuh, Malte Hellwig, Judith Niesen, Volker Mall, Birgit Ertl-Wagner, Christian Hagel, Michael Spohn, Beat Lutz, Jan Sedlacik, Daniela Indenbirken, Daniel J. Merk, Ulrich Schüller

**Affiliations:** 1 Research Institute Children’s Cancer Center Hamburg, Martinistrasse 52, N63 (HPI), 20251 Hamburg, Germany; 2 Department of Pediatric Hematology and Oncology, University Medical Center, Hamburg-Eppendorf, 20246 Hamburg, Germany; 3 Center for Neuropathology, Ludwig-Maximilians-University, 81377 Munich, Germany; 4 Department of Pediatric Oncology and Hematology, Charité University Medical Center, 13353 Berlin, Germany; 5 German Center for Neurodegenerative Diseases (DZNE), Ludwig-Maximilians-University, 81377 Munich, Germany; 6 Department of Pediatrics, Technical University of Munich, kbo Children’s Centre, 81377 Munich, Germany; 7 Institute for Clinical Radiology, Ludwig-Maximilians-University, 81377 Munich, Germany; 8 Division of Neuroradiology, The Hospital for Sick Children, University of Toronto, Toronto, Canada; 9 Institute of Neuropathology, University Medical Center, Hamburg-Eppendorf, 20246 Hamburg, Germany; 10 Bioinformatics Facility, University Medical Center, Hamburg-Eppendorf, 20246 Hamburg, Germany; 11 Institute of Physiological Chemistry, University Medical Center of the Johannes Gutenberg University, 55128 Mainz, Germany; 12 Department of Neuroradiological Diagnostics and Intervention, University Medical Center, Hamburg-Eppendorf, 20246 Hamburg, Germany; 13 Heinrich-Pette-Institute, Leibniz Institute for Experimental Virology, 20251 Hamburg, Germany; 14 Hertie Institute for Clinical Brain Research, University Hospital Tübingen, 72076 Tübingen, Germany

**Keywords:** Creb binding protein (CREBBP, CBP), Rubinstein-Taybi syndrome (RSTS), Neural precursor cell migration, Adult neurogenesis, Neural differentiation

## Abstract

CREB (cyclic AMP response element binding protein) binding protein (CBP, CREBBP) is a ubiquitously expressed transcription coactivator with intrinsic histone acetyltransferase (KAT) activity. Germline mutations within the *CBP* gene are known to cause Rubinstein-Taybi syndrome (RSTS), a developmental disorder characterized by intellectual disability, specific facial features and physical anomalies. Here, we investigate mechanisms of CBP function during brain development in order to elucidate morphological and functional mechanisms underlying the development of RSTS. Due to the embryonic lethality of conventional CBP knockout mice, we employed a tissue specific knockout mouse model (*hGFAP-cre::CBP*^*Fl/Fl*^, mutant mouse) to achieve a homozygous deletion of CBP in neural precursor cells of the central nervous system.

Our findings suggest that CBP plays a central role in brain size regulation, correct neural cell differentiation and neural precursor cell migration. We provide evidence that CBP is both important for stem cell viability within the ventricular germinal zone during embryonic development and for unhindered establishment of adult neurogenesis. Prominent histological findings in adult animals include a significantly smaller hippocampus with fewer neural stem cells. In the subventricular zone, we observe large cell aggregations at the beginning of the rostral migratory stream due to a migration deficit caused by impaired attraction from the CBP-deficient olfactory bulb. The cerebral cortex of mutant mice is characterized by a shorter dendrite length, a diminished spine number, and a relatively decreased number of mature spines as well as a reduced number of synapses.

In conclusion, we provide evidence that CBP is important for neurogenesis, shaping neuronal morphology, neural connectivity and that it is involved in neuronal cell migration. These findings may help to understand the molecular basis of intellectual disability in RSTS patients and may be employed to establish treatment options to improve patients’ quality of life.

## Introduction

Rubinstein-Taybi syndrome (RTS or RSTS; OMIM #180,849, #613,684), first described in 1963, is a rare but archetypal developmental disorder with multiple congenital anomalies, intellectual disability and a prevalence of 1:100,000 to 1:125,000 at birth [22, 23, 48]. It is characterized by microcephaly and intellectual disability, postnatal growth impairment, specific facial abnormalities and broad, angulated thumbs and big halluces [9]. Genetic analyses uncovered heterozygous mutations in the highly homologous *CREB binding protein (CBP)* and *p300* genes (also called *EP300* or *E1A binding protein p300*) genes to be causative for the development of RSTS [48, 53]. Mutations of *CBP* can be found in 40–60% of patients with RSTS and mutations of *p300* are observed in approximately 10% of cases [52]. Though showing an autosomal dominant character, RSTS is mostly caused by de novo mutations. The causative mutations in the *CBP* gene include point mutations, small deletions and duplications, which may lead to premature translational stops as well as large deletions, including *CBP* and flanking genes [14, 36, 38, 48]. CBP, as well as its homolog p300, is a ubiquitously expressed transcriptional coactivator known to play an important role in embryonic development, growth control and cell homeostasis [20]. It has an intrinsic lysine acetyltransferase (KAT) activity and stabilizes protein interactions with the transcription complex, thus mediating chromatin remodeling and transcription factor recognition [31, 44]. It was shown to integrate signals from a multitude of signaling pathways, interacting with more than 400 transcription factors and other regulatory proteins, and to be present at promoters of more than 16,000 human genes [5, 50]. Mouse models for RSTS with a conventional global heterozygous loss of CBP have been established and delivered indications for the causative role of the heterozygous loss of CBP or its KAT activity [4, 33, 45, 59]. However, despite the heterozygous loss being the appropriate resemblance of the human situation, conventional global heterozygous knockout mouse models did not help to explain the intellectual disability in RSTS patients [4, 62].

The knock-in and conditional knock-out models generated so far contributed largely to the existing knowledge about the mechanisms behind RSTS [15, 27, 62]. The models include for example a knockout in postmitotic neurons as well as a central nervous system (CNS)-specific knockout and reveal an effect on long-term memory formation in CBP-deficient postmitotic neurons and effects of CBP on neuron morphology.

Thus, although a cognitive deficit was observed in all mouse models generated so far, the mechanisms underlying the intellectual disability could not be determined. Therefore, the effects of a complete loss of CBP during embryonic development in a CNS-specific conditional homozygous CBP knockout mouse model driven by the human glial fibrillary acidic protein (hGFAP) promoter (*hGFAP-cre::CBP*^*Fl/Fl*^) were studied both in vivo and in vitro. A focus was thereby placed on the analysis of development and integrity of the forebrain structures neocortex, hippocampus, and olfactory bulb (OB). Additionally, postnatal neurogenesis and developmental processes such as neural precursor cell (NPC) proliferation and viability, neural differentiation and precursor cell migration were investigated.

Using this homozygous knockout approach, we were able to demonstrate that CBP function is crucial for proper brain development, cell differentiation and NPC migration as well as establishment of adult neurogenesis.

## Results

### *hGFAP-cre::CBP*^*Fl/Fl*^ mice resemble aspects of human RSTS patients like microcephaly and behavioral anomalies

**Fig. 1:**
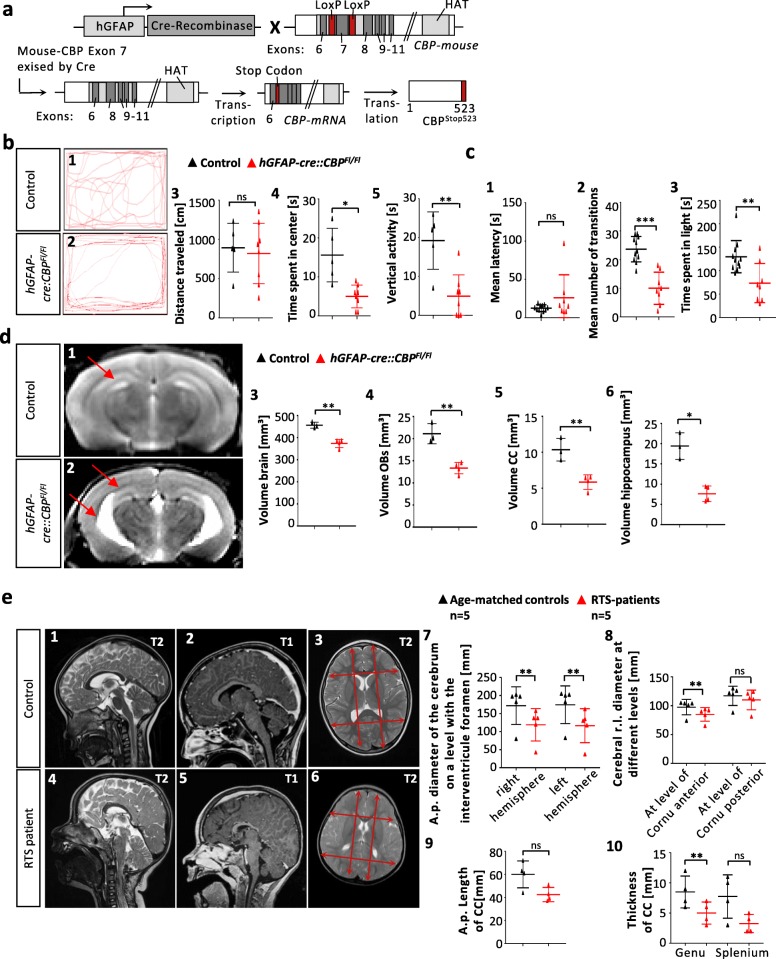
*hGFAP-cre::CBP*^*Fl/Fl*^ CBP deficient mice exhibit abnormal behavior and a decrease in brain volume corresponding well with findings in brain MR images from RSTS patients. **a** In *hGFAP-cre::CBP*^*Fl/Fl*^ mice, CBP^Stop523^ is expressed in cells that express the cre-recombinase under the *hGFAP* promoter. CBP^Stop523^ does not contain the KAT domain. LoxP: Cre-recombinase recognition and incision site, KAT: Lysine acetyltransferase domain. **b1**–5 Open field test: tracked mouse movements in the open field test for one representative control and mutant animal. Movement analysis shows that transgenic mice spend significantly less time in the center and show significantly less vertical activity. **c1**–**3** Dark/light box test: *hGFAP-cre::CBP*^*Fl/Fl*^ mice spent significantly less time exploring the bright chamber and transitioned less often between the two compartments. **d1**–**6** Representative coronal T2 weighted brain MR images of transgenic and control animals with arrows marking the hypoplastic hippocampus and the widened lateral ventricle in the mutant. Volumetric analysis unveiled significantly reduced brain volume, OB size, CC volume and hippocampus size after early loss of CBP. **e1-6** Sagittal T2 and T1 weighted and axial T2 weighted MR images of a RSTS patient and age-matched control child. Microcephaly and a smaller brain are noted for the RSTS patient. CC length- and thickness measurements as well as anterior-posterior & left-right diameter measurements displayed as red arrows.**e7-8** Paramedian anterior-posterior diameter and the anterior left-right diameter were significantly reduced in RSTS patients. **e9-10** Thickness of the CC genu is significantly reduced in RSTS patients. *p < 0.05, **p < 0.01, ***p < 0.001

To assess the impact of the CBP knockout specifically in the developing brain, *hGFAP-cre* and *CBP*^*Fl/Fl*^ mice were mated to generate *hGFAP-cre::CBP*^*Fl/Fl*^ transgenic mice. In this homozygous conditional knockout model, the loxP flanked (floxed) CBP-coding gene is knocked out in cells expressing hGFAP. This accounts mainly for NPCs from embryonic day (E) 12.5 - E13.5 onwards and for astrocytes of the mouse brain [8, 75]. After recombination, the cells express a C-terminally truncated version of the mouse CBP, *CBP*^*Stop523*^, which lacks 24 of its 31 exons including the KAT domain (Fig. 1a). To further validate this knockout model as a suitable animal model for RSTS, the major role of diminished KAT activity in RSTS pathogenesis was verified through mutation distribution analysis. We analyzed published RSTS cases as listed in the Human Gene Mutation Database (Qiagen Bioinformatics) with a focus on KAT domain mutations. In line with previous findings**,** we show that RSTS-causing mutations were not distributed equally within the *CBP* gene (Additional file 1: Figure S1a1) [22, 26, 48, 52]. Instead, significantly more pathogenic point mutations are reported within the KAT domain than in other regions (Additional file 1: Figure S1a2). Furthermore, nonsense mutations in exon 1–17 – leading to a loss of the KAT domain – were significantly more frequent than missense mutations in the same region, also supporting the essential function of this domain (Additional file 1: Figure S1a4).

We first analyzed the behavior of the *hGFAP-cre::CBP*^*Fl/Fl*^ transgenic mice through an open field (Fig. 1b) and a dark/light box (Fig. 1c) test, which suggested a shifted anxiety/curiosity ratio towards more aversive behavior compared to control animals (animals without cre recombinase). In the open field test, mutants travelled a similar distance as compared to their wild type littermates (Fig. 1c3) but explored the open field to a lesser extent (Fig. 1b1,2,4,5).

The animals entered the bright chamber significantly less often (Fig. 1c2) and spent less time in the light (Fig. 1c3).

The volume and weight of the adult brain of *hGFAP-cre::CBP*^*Fl/Fl*^ mice was significantly reduced (Fig. 1d3, Additional file 1: Figure S1b). Most major brain regions were affected by the CBP dependent reduction in size – e.g. the OB (Fig. 1d4), the corpus callosum (CC) (Fig. 1d5) and the hippocampus (Fig. 1d6) as demonstrated and measured in Magnetic Resonance (MR) images of the mice. This corresponds well with the conducted case-control study on brain MR images from children with genetically confirmed diagnosis of RSTS compared to age-matched controls. It revealed significantly reduced anterior-posterior diameters of both hemispheres in axial sections (Fig. 1 e3,6,7,8). Additionally, the CC was shorter and the genu was significantly thinner (Fig. 1e1,4,9,10). Myelination deficits and dysmorphic heads were found in the MR images of RSTS patients. Also, the distance between the base of the frontal lobe to the sellar floor was significantly greater in the MR images of RSTS patients, which fits to the reduced brain sizes (Additional file 1: Figure S1c1–3). Width measurements of the anterior and posterior horns of the lateral ventricles, however, did not show differences between RSTS patients and control children (Additional file 1: Figure S1c4). These findings confirmed the mostly qualitative description of microcephaly, smaller brain sizes and CC dysplasia in RSTS patients [1, 6, 34, 41, 69, 70].

### A tissue specific, early loss of CBP leads to structural alterations in the developing forebrain with impaired postnatal neurogenesis in the hippocampus and the olfactory bulb

**Fig. 2:**
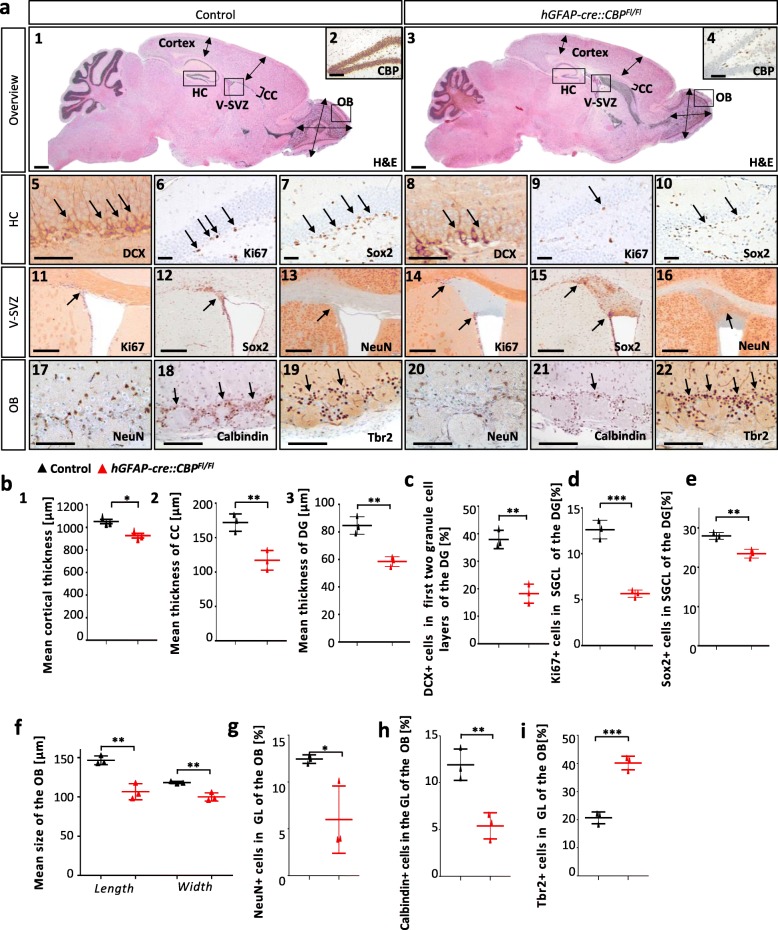
A tissue specific, early loss of CBP leads to structural alterations in the developing forebrain with impaired postnatal neurogenesis in the hippocampus and OB. **a1,3** Sagittal H&E stained sections through the RMS at P30. Boxes mark the areas that were investigated subsequently. Arrows indicate representative size measurements of the neocortex thickness and OB width and length. Square brackets mark the CC thickness. **b**, **f** Mean neocortical thickness, CC thickness, dentate gyrus thickness as well as OB width and length are significantly smaller in *hGFAP-cre::CBP*^*Fl/Fl*^ animals aged 30 days. **a2**, **a4** CBP stained regions of the hippocampus and neocortex displaying the reduction of CBP expression in the mouse model. **a5**–**10** DCX staining, Ki67 staining and Sox2 staining of frontal sections through the dentate gyrus. **c**, **d**, **e** DCX+, Ki67+ and Sox2+ rates were significantly reduced in the SGZ of the dentate gyrus of *hGFAP-cre::CBP*^*Fl/Fl*^ mice at P30. **a11**-**a16** Ki67, Sox2 and NeuN stained frontal sections through the left lateral ventricle and V-SVZ and the cell accumulation in mutant animals. In Ki67 stained sections V-SVZ typical areas are marked by arrows. Sox2 and NeuN stained frontal sections show different cytoarchitectural areas within the accumulation. **a17**–**22** NeuN neuronal staining, Calbindin staining of GABAergic periglomerular neurons and Tbr2 staining of glutamatergic juxtaglomerular neurons in the glomerular layer (GL) at P30. **g** Significantly lower rate of NeuN positive cells after early loss of CBP in the GL. **h**, **i** Whereas the rate of Calbindin positive inhibitory neurons is shown to be significantly reduced in the GL of the OB in *hGFAP-cre::CBP*^*Fl/Fl*^ mice, the rate of Tbr2 positive excitatory neurons is significantly increased compared to control animals. *Scale bar: 2000* μm (a 1, 3), 200 μm (a 2, 4), 50 μm (a 5–10, 17–22), 150 μm (a 11–16); *p < 0.05, **p < 0.01, ***p < 0.001

Besides the size reduction, structural abnormalities were found in different brain regions in *hGFAP-cre::CBP*^*FlFl*^ mice. We focused our analysis on the forebrain and found alterations in the hippocampus, the ventricular-subventricular zone (V-SVZ) and the OB (Fig. 2a). The general development of the different hippocampal zones cornu ammonis (CA) I-III and the dentate gyrus was not found to be hindered (Additional file 1: Figure S2a). However, we observed a reduction of postnatal neurogenesis in the hippocampal subgranular zone (SGZ) at postnatal day (P) 30, indicated by the reduction of NPC numbers- (DCX+ and Sox2+ cells) and proliferation rates (Ki67+) (Fig. 2a5–10,c,d,e). No apoptosis (cleaved Caspase 3+ cells) was detected in the analyzed areas (Additional file 1: Figure S2a).

Furthermore, a cell accumulation at the beginning of the rostral migratory stream (RMS) close to the V-SVZ and below the CC was visible only in mutants (Fig. 2a3,14,15,16). Within the cell aggregation, two different cell populations were observed. One cell population was marked by Sox2 and Ki67 expression and resembled a more premature, proliferating population, typically found at the border of the V-SVZ (Fig. 2a11,12,14,15). In contrast, another population contained NeuN+ cells, which indicates a differentiation of cells into neurons (Fig. 2a,16). This suggested that a certain fraction of V-SVZ neuroblasts accumulated at the border of the V-SVZ instead of travelling to the OB and at least partly differentiated into mature neurons. Within this cell accumulation, apoptotic cells (cleaved Caspase 3+), were present (Additional file 1: Figure S2b). A neoplastic process appeared unlikely, as Ki67 positivity only remained in V-SVZ typical areas and was scarce in most areas within the accumulation (Fig. 2a11,14).

Since we hypothesized that NPCs do not migrate properly into the OB, we analyzed structural differences in the OB. Neither the internal plexiform layer (IPL) nor the RMS bundle within the OB were consistently determinable in the mutant mice (Fig. 2a1,3, Additional file 1: Figure S2d). NeuN was used as a neuronal marker to further investigate the composition and integrity of the different layers. It was shown that in both, the granule cell layer (GCL) as well as the glomerular layer (GL), showed a reduced number of NeuN positive neuronal cells in *hGFAP-cre::CBP*^*Fl/Fl*^ animals (Fig. 2a17,20,2g, Additional file 1: Figure S2d5,6,13). Cell subpopulation composition and excitatory-inhibitory balance in the GL were investigated further using Tbr2 (excitatory neurons) and Calbindin (inhibitory neurons) stains [17, 32, 42, 43, 47]. We found a significantly reduced percentage of Calbindin+ neurons (Fig. 2a18,21,2h) accompanied by an increase of Tbr2+ cells in the GL of CBP deficient mice (Fig. 2a19,22,2i). In this regard, olfactory related behaviour was shown to be affected by early loss of CBP in a buried food test (BFT) and habituation/dishabituation test, uncovering reduced olfactory detection ability in the mutant animals (Additional file 1: Figure S2e).

Another important anatomical structure in memory processing is the neocortex. Here, basic cytoarchitecture, proliferation, apoptosis as well as neural differentiation were determined. We demonstrate that apart from a significantly thinner cortex (Fig. 2b1), the general establishment of the six neocortical layers as well as proliferation and apoptosis were unaffected by the CBP depletion (Additional file 1: Figure S2f). In terms of myelination, no overt differences were identified between control and mutant animals (Additional file 1: Figure S2g).

### During embryonic development a homozygous CBP knockout in NPCs induces apoptosis and leads to reduced proliferation in the ventricular zone

**Fig. 3:**
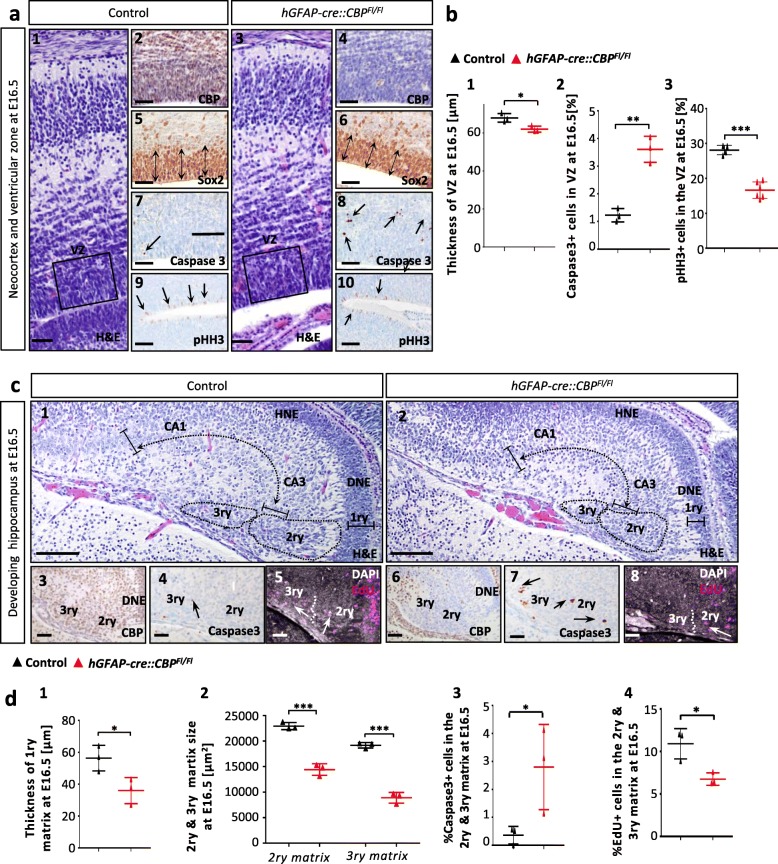
During embryonic development, homozygous CBP knockout in NPCs induces apoptosis and leads to reduced proliferation in the ventricular zone. **a1**–**10** Overview of ventricular zone and the emerging neocortex at E16.5 in H&E staining of frontal sections with confirmation of CBP knockout in transgenic animals. Sox2 marks stem cells, pHH3 proliferating cells and Caspase3 apoptotic cells in high-power magnifications of the ventricular zone at E16.5. **b1**–**3** Ventricular zone thickness significantly reduced at E16.5 in transgenic mice. A significant rise in apoptosis rate and a significantly diminished proliferation rate was measured within the ventricular zone of transgenic mice. **c1**–**8** Overview of hippocampal formation at E16.5 in H&E staining of frontal sections. Displaying the Cornu ammonis (CA1 and CA3), hippocampal neuroepithelium (HNE), dentate neuroepithelium (DNE, 1ry) and 2ry & 3ry matrix, with a complete CBP depletion in both the DNE and in migrating cells in knockout animals. High power magnifications of the 2ry & 3ry matrix of the developing dentate gyrus at E16.5 with Caspase3 apoptosis staining and EdU proliferation with DAPI nucleus staining (EdU injection at E16.5). Black arrows mark Caspase3+ apoptotic cells and white arrows EdU+ proliferating cells. **d1**–**2** 1ry matrix (DNE), significantly thinner and area sizes of 2ry & 3ry matrix, significantly reduced in *hGFAP-cre::CBP*^*Fl/Fl*^ animals at E16.5. **d3**–**4** Significant increase in apoptosis rates and significant decrease in proliferation in the 2ry and 3ry matrix of *hGFAP-cre::CBP*^*Fl/Fl*^ mice at E16.5 c. Scale bar: 80 μm (a1,3) 50 μm (a2,4–10, c3–8), 100 μm (c1,2); *p < 0.05, **p < 0.01, ***p < 0.001

Brain development was investigated at E14.5 and E16.5 in CBP KO mice. We first analyzed the neocortex of *hGFAP-cre::CBP*^*Fl/Fl*^-mice. In the neocortex, neurogenesis starts at E10.5 and lasts until E18 [61, 63]. First, after confirmation of CBP knockout the pluripotent neural stem cell (NSC) character of cells in the ventricular zone (VZ) was verified by Sox2 staining (Fig. 3a1-6) [60, 74]. The VZ of transgenic animals was significantly thinner at E16.5 (Fig. 3a5,6,b1), which is in line with a reduced proliferation (visualized by Bromodeoxyuridine (BrdU) staining; Injection 2 h before sacrifice) at E14.5 (Additional file 1: Figure S3d3,7,9) and E16.5 (Fig. 3a9,10,b3). The apoptosis rate was significantly increased in the VZ of *hGFAP-cre::CBP*^*Fl/Fl*^ mice at E16.5 (Fig. 3a7,8,b2), whereas no difference was found at E14.5 (Additional file 1: Figure S3d2,6). No differences in proliferation or apoptosis were observed within the neocortical cell layers (Additional file 1: Figure S3c).

Investigation of hippocampus- and dentate gyrus development was also conducted. The development of the granule cell lineage of the dentate gyrus was unaffected by the CBP deletion, demonstrated by using Sox2 and Prox1 stains (expressed in dentate gyrus granule cells) (Additional file 1: Figure S3e7–10) [46]. Additionally, structural differences were analyzed, and a significantly reduced thickness of the 1ry matrix was measured at E16.5 (Fig. 3c1,2,d1), whereas at E14.5 no differences were observed (Additional file 1: Figure S3f1). Furthermore, area measurements in frontal sections confirmed significantly smaller 2ry and 3ry matrices at E16.5 (Fig. 3c1,2,d2). Cell viability and proliferation were assessed at both of these embryonic stages in the developing hippocampus. At E14.5, the proliferation rate in the Dentate neuroepithelium (DNE) was significantly reduced, whereas no proliferation was seen among the migrating cells in the 2ry matrix and no relevant apoptosis was observable in the DNE and the 2ry matrix (Additional file 1: Figure S3e11–14, f2,3). Analysis of apoptosis and proliferation rates at E16.5 showed stronger effects of the CBP depletion: Apoptosis was detectable in the 1ry matrix of *hGFAP-cre::CBP*^*Fl/Fl*^ animals, and the apoptosis rate was significantly increased in the area of 2ry and 3ry matrix of mutant mice (Fig. 3c4,7,d3). Furthermore, the proliferation in the 2ry and 3ry matrix (visualized by 5-Ethynyl-2′-deoxyuridine (EdU) staining; Injection 2 h before sacrifice) was significantly reduced in *hGFAP-cre::CBP*^*Fl/Fl*^ animals (Fig. 3c5,8,d4). Together, these findings suggest that the structural alterations of the hippocampus found in postnatal mice root in a disturbed prenatal development with reduced proliferation and NPC viability especially in the VZ.

### Neocortical layer V pyramidal cells lacking CBP show reduced volumes and alterations in spine density and morphology

**Fig. 4:**
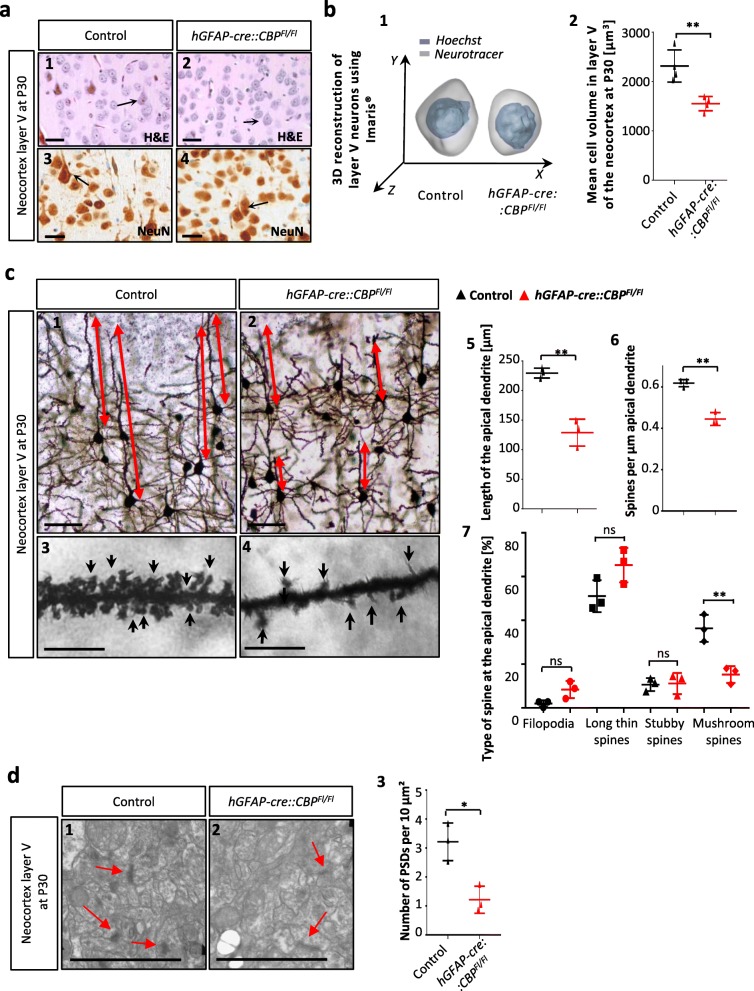
Neocortical layer V pyramidal cells show reduced volumes and alterations in spine density & morphology when missing CBP. **a1**–**4** Neural differentiation in layer V of the neocortex, made visible through NeuN staining. Arrows mark lamina V giant pyramidal cells that appeared to be smaller and dysmorphic in transgenic mice. **b1** 3D cell reconstruction of Lamina V giant pyramidal cells from Hoechst and neurotracer stained 100 μm sections (**b2**) Determined mean cell volume in the lamina V of the neocortex at P30, significantly smaller in the transgenic mice compared to control litter mates. **c1**–**4** Golgi-Cox impregnated pyramidal neurons in layer V show severe abnormalities and maturation deficits in *hGFAP-cre::CBP*^*Fl/Fl*^ mice. **c5**,**6** The apical dendrite is significantly shorter and less dendritic spines can be observed. **c7** The spine type distribution is shifted towards less mature spines. **d1**–**3** Analysis in electron microscopic images of layer V neurons show that the number of synapses was significantly diminished in the cerebral cortex of *hGFAP-cre::CBP*^*Fl/Fl*^ mice. Scale bar: 50 μm (a1–4), 100 μm (c1–2), 10 μm (c3–4), 5 μm (d1–2); *p < 0.05, **p < 0.01

The neocortical structure as well as the density of mature neurons was not found to be severely altered in mutant mice, but the neurons appeared to be smaller and dysmorphic especially in lamina V (Fig. 4a). To shed more light on the influence of early loss of CBP on the morphology of neocortical neurons on a cellular level, 3D reconstruction of lamina V giant pyramidal cells was conducted. Neurons without a functional CBP showed a significantly lower cell volume (Fig. 4b). This can also be seen in volume distribution curves for the two groups, which displayed a shift of the curve towards smaller volumes in the mutant mice (Additional file 1: Figure S4a).

To investigate the morphological changes of neurons upon CBP deletion further, we employed Golgi-Cox staining to visualize the entire cell body including dendrites and spines. First, we determined the length of the apical dendrite of layer V giant pyramidal neurons in order to assess whether loss of CBP affects cellular morphology beyond the cell soma. We showed that the apical dendrites of neurons without a functional CBP were significantly shorter compared to their wild type counterparts. In average, the dendrite length in the mutants was reduced by 50% suggesting functional consequences for the neuron (Fig. 4c1,2,5). The number of branches per μm dendrite was not altered (Additional file 1: Figure S4b). Additionally, we aimed at investigating neuron morphology and neuronal functions further and therefore analyzed dendritic spines. We found the number of spines per μm apical dendrite to be significantly reduced by one third in the mutant cortex (Fig. 4c3,4,6).

As previously described, four different types of neuronal spines exist in mature neurons which are believed to represent different maturation stages and dynamics. Thereby, the more mature stages, stubby and mushrooms spines, are thought to be more stable whereas filopodia and long thin spines are more dynamic [7]. We observed a disturbed balance of the spine types with a shift towards more immature spines in *hGFAP-cre::CBP*^*Fl/Fl*^ animals. Mushroom spines, being the most differentiated spine type, were significantly reduced in the mutant cortex whereas the relative amount of more immature types - filopodia and long thin spines - was increased (Fig. 4c3,4,7).

In order to get a more detailed view on pyramidal neurons in the cortex and especially its corresponding synapses, we used electron microscopy. We showed that the number of post synaptic densities (PSDs) was reduced by two thirds in CBP deficient animals which complemented our previous observations (Fig. 4d). In comparison, the length of the existing PSDs was unaffected by the CBP deletion (data not shown). This implicates that neural differentiation and development is disturbed in NPCs lacking CBP.

### NPC migration is compromised upon CBP knockout, both pre- and postnatally

**Fig. 5:**
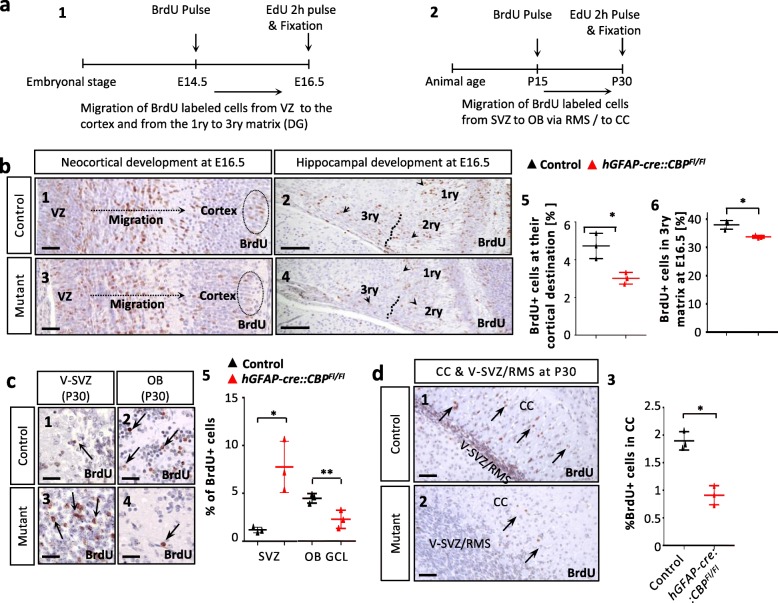
Neural precursor cell migration is compromised when CBP is knocked out at an early time-point in vivo. **a1**,**2** BrdU/EdU fate mapping experimental design. For analysis of embryonic development, BrdU was injected at E14.5 labeling proliferating and then migrating cells within the ventricular zone. At E16.5, animals were sacrificed after EdU administration for marking currently proliferating cells. For analysis of postnatal migration from the V-SVZ towards the corpus callosum and OB through the RMS, BrdU was administered at P15 and EdU at P30. **b1**,**3** BrdU fate-mapping in frontal sections from the ventricular zone towards the outer neocortical layers, which are marked with circles at E16.5. **b2**,**4** BrdU fate-mapping in frontal sections at E16.5 displaying the developing hippocampus with 1ry, 2ry and 3ry matrix. **b5** Percentage of migrating BrdU+ cells that reached the circled outer layers of the neocortex. In mutant animals, significantly less cells reached their destination. **b6** Percentage of migrating BrdU+ cells that reached the 3ry matrix compared to all BrdU+ cells, with a significantly smaller proportion of BrdU+ cells reaching the 3ry matrix after loss of CBP. **c1**,**3** Frontal sections through V-SVZ at P30 with arrows showing BrdU+ cells within the V-SVZ of control animals and within the cell accumulation seen in *hGFAP-cre::CBP*^*Fl/Fl*^ mice. **c2**,**4** Frontal sections through the OB at P30, showing BrdU+ cells after their migration through the RMS (arrows). **c5** The BrdU+ rate was found to be significantly higher within the cell accumulation than in the V-SVZ of control animals and significantly lower in the GCL of the OB of transgenic animals. **d1**,**2** BrdU staining of the CC genu and V-SVZ/RMS in sagittal sections at P30 after BrdU injection at P15. Migrated BrdU+ cells in the CC are labelled with arrows. **d3** BrdU+ rate in the corpus callosum at P30, significantly lower in *hGFAP-cre::CBP*^*Fl/Fl*^ mice. Scale bar: 50 μm (b1–4), 30 μm (c1–4); 100 μm (d1,2); *p < 0.05, **p < 0.01

BrdU/EdU pulse-chase experiments were used to investigate the migration of NPCs in vivo. For assessing the migration of NPCs during neocortical development, proliferating cells in the VZ were marked with BrdU at E14.5 and the analysis was conducted at E16.5 after those cells traveled radially to form the neocortical layers (Fig. 5a1). We found that the percentage of migrating BrdU+ cells which reached their destination, the outer neocortical layers, were significantly reduced in the mutant animals (Fig. 5b1,3,5).

Additionally, the migration of cells in the developing hippocampus was investigated in the same pulse-chase experiment. Between E14.5 and E16.5 NPCs migrate from the 1ry to the 2ry and 3ry matrix to contribute to dentate gyrus formation. The migration of NPCs was impaired when lacking a functional CBP as significantly fewer cells reached the 3ry matrix in the mutant animals compared to the situation in wild type animals (Fig. 5b2,4,6).

Additional to the investigation of prenatal NPC migration, we also looked further into postnatal migration processes. To confirm that a migration deficit was the cause of the observed cell accumulation and the reduced number of NeuN positive cells in the OB found at P30 (Fig. 2), these structures were analyzed in a P15-P30 fate-mapping experiment (Fig. 5a2). The 15-day interval was chosen, as neuroblasts generated in the V-SVZ take approximately 10–14 days to travel through the RMS to their destination in the OB [10]. The percentage of BrdU+ cells was determined in the V-SVZ of control animals, the cell accumulation of transgenic mice and in the GCL and GL of the OB of all animals (Fig. 5c, Additional file 1: Figure S5a). Whereas the BrdU+ rate in the cell accumulation of *hGFAP-cre::CBP*^*Fl/Fl*^ mice was significantly higher than in the V-SVZ of control mice, the BrdU+ rate in the GCL of the mutant OB was significantly lower (Fig. 5c). At the same time, no cells were found to be both BrdU+ and EdU+ and thus still proliferating regardless of the genotype. Hence, the observed differences in BrdU+ rates in the different areas were not to be explained by differences in continued proliferation of the neuroblasts once they left the V-SVZ (Additional file 1: Figure S5a2,4–6). In the GL only a tendency towards lower BrdU+ rates in *hGFAP-cre::CBP*^*Fl/Fl*^ mice was observed. However, by normalization for the reduced NeuN+ rate in the GL, a significant reduction of neurogenesis could be shown here as well (Additional file 1: Figure S5b). These findings implicate a migration deficit that leads to the cell accumulation and the underdeveloped OB.

Additionally, we investigated the postnatal migration of NPCs into the CC. It was described that astroglial progenitor cells travel from the V-SVZ to the CC in the adult organism [58]. To investigate this process in *hGFAP-cre::CBP*^*Fl/Fl*^ mice, BrdU was injected at P15 and analysis was performed 15 days later (Fig. 5a2). At P30, mutant mice displayed a significantly reduced percentage of BrdU+ cells in the genu of the CC compared to the control group (Fig. 5d). No differences in proliferation or apoptosis within the V-SVZ or in the CC at P15 and P30 were observed, suggesting that cell migration from the V-SVZ to the CC is impaired (Additional file 1: Figures S2c and S5c).

### CBP regulates NPC migration through an extrinsic protein secreted by the OB

**Fig. 6:**
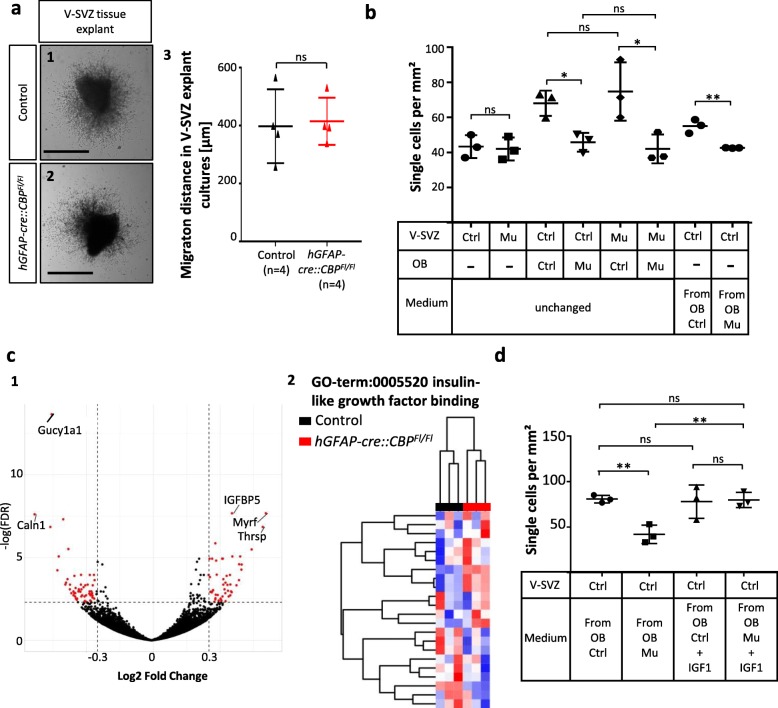
The migration deficit observed in *hGFAP-cre::CBP*^*Fl/Fl*^ mice is mediated by an IGF1-deficiency in the olfactory bulb. **a1**–**3** The intrinsic migration potential of SVZ derived neuroblasts is independent of CBP function, shown through the culture of SVZ explants in the extracellular matrix Matrigel. **b** The number of single cells was measured around explants of the SVZ cultured in Matrigel. A piece of OB from the wild type induces an exit of neuroblasts from their migratory chains and thereby an increase in single cells. The mutant OB is unable to induce this effect. The same effect can be observed with medium conditioned with tissue from the OB of wild type or mutant mice. **c1** RNA Sequencing (Seq.) of SVZ explants, which were stimulated with medium conditioned from the OB of control or *hGFAP-cre::CBP*^*Fl/Fl*^. In total, 106 genes are statistically different between control and mutant with a log2-fold change ≥0.25 and an FDR < 0.1. Genes with high log2-fold changes are annotated. **c2** RNA Seq. reveals a difference in IGF signaling between control and mutant. Clustering of RNA Seq. data by the GO-term “0005520 insulin-like growth factor binding” reveals clustering according to the genotype. (**d**) IGF1 is able to rescue the effect of the CBP deletion on the exit of neuroblasts from chain migration. The addition of recombinant IGF1 to medium conditioned by the OB of control mice has no effect on the number of single cells. In contrast, the number of single cells in the presence of medium conditioned by a CBP deficient OB tissue supplemented with IGF1 is comparable to the number of single cells from wild type medium. Scale bar: 1 mm; *p < 0.05, **p < 0.01

To elucidate the exact mechanisms how CBP influenced migration, we used an in vitro approach. It was first described in 1997 that tissue explants of the V-SVZ can be cultivated and that chains of neurons migrate out of the explants, recapitulating the situation in vivo [68]. This experiment is used to investigate the intrinsic migration ability of cells. It had been shown before that the knockout of different genes can impair the migration potential of neuroblasts [11, 12, 21, 29, 66]. We hypothesized from the in vivo data, that the loss of CBP might have a similar effect. However, using this approach, we did not observe a difference in the migration of NPCs after loss of CBP function (Fig. 6a).

As this suggested that the intrinsic migration ability of the NPCs was not affected by the CBP deletion, we hypothesized that extrinsic factors might be responsible for the observed effect in vivo. To test this hypothesis, we co-cultured tissue explants of the V-SVZ with tissue of the OB of control and *hGFAP-cre::CBP*^*Fl/Fl*^ mice and analyzed the number of single cells leaving the explant. Usually, the presence of OB tissue induces a change of migration mode in neuroblasts from the tangential chain migration to an individual radial migration, recapitulating the situation in vivo [21]. Using this experimental set-up we detected a significant effect of the CBP deletion on cell migration. In a co-culture with tissue of *hGFAP-cre::CBP*^*Fl/Fl*^ OB, cells from the V-SVZ tissue explant of either control or *hGFAP-cre::CBP*^*Fl/Fl*^ mice exhibited a significantly reduced number of single cells surrounding the tissue explant as compared to the co-culture with control OBs (Fig. 6b).

In order to investigate if a secreted factor from the OB is responsible for the observed migration deficit of neuroblasts, we employed another experiment with tissue explants of the V-SVZ. In this experiment, we used OB-conditioned medium of control and *hGFAP-cre::CBP*^*Fl/Fl*^ mice and assessed the number of single cells leaving the V-SVZ explant (Fig. 6b). We showed that the conditioned medium from the mutant OB was able to produce the same effect of a reduction of single cells as the tissue of the mutant OB itself, suggesting that the molecule responsible for the described migration deficit is a soluble factor. In an attempt to identify this factor we performed RNA Sequencing of stimulated V-SVZ tissue explants. In total, the expression of 106 genes was statistically different between V-SVZ explants stimulated with medium from a wild type OB explant compared with V-SVZ explants stimulated with medium from a CBP deficient OB (Fig. 6c1). One of the most upregulated genes was *IGFbp5* (Fig. 6c1), which we focused on as it was described that IGF1 (Insulin-Like Growth Factor IB9) plays a role in NPC migration [24]. We therefore analyzed our RNA Sequencing data for genes, which are involved in IGF signalling (GO term 0005520 insulin-like growth factor binding). When we clustered the results of the RNA Sequencing data for those genes, we saw that the control and the mutant form two distinct clusters (Fig. 6c2). We then compared the phenotype of IGF1- and our CBP knockout mice in order to find further hints that IGF signalling might be involved in the migration deficiency in our mouse model. IGF1 knockout mice, described by Hurtado-Chong et al. in 2007, have smaller OBs and a cell accumulation at the V-SVZ, which is caused by the inability of NPCs to leave the RMS [24]. Using the indications generated from the RNA Sequencing data and the comparison of mouse knock out studies, we employed our in vitro migration assay and tested, if we could rescue the migration deficit by addition of recombinant IGF1 to the conditioned medium from the OB. As shown in Fig. 6d, IGF1 was indeed able to rescue the effect of the CBP knockout on NPC migration, whereas the migration of wild type cells was unaffected by the IGF1 treatment.

## Discussion

### A homozygous knockout model for a disease with a heterozygous genotype

In this study, employing a CNS specific depletion of CBP, we provide evidence that CBP is important for the establishment of neuronal morphology and migration. This contributes to the knowledge about CBP protein function especially in the context of neurodevelopment. We decided to use a tissue specific knockout mouse model for studying this neurodevelopmental disorder for a number of reasons. First, even though conventional heterozygous mouse models mimic the situation in human RSTS patients genetically and phenotypically, the role of CBP in forebrain development and RSTS related cognitive impairment was constrained by partial and uncertain levels of CBP function inhibition [4]. Furthermore, conventional homozygous CBP knockout mice die prenatally [33, 45]. Hence, a homozygous tissue-specific CBP knockout model, *hGFAP-cre::CBP*^*Fl/Fl*^, was used to study the role of CBP in forebrain development more profoundly and to identify developmental processes that depend highly on CBP and may be impaired in RSTS.

Abnormal findings on MR imaging including microcephaly, cerebellar hypoplasia and a reduced CC size were observed both in the used mouse model and in the cranial MR images of RSTS patients and congruent to the published literature [1, 3, 34]. Cerebellar hypoplasia and structural abnormalities in the cerebellum that were detected in *hGFAP-cre::CBP*^*Fl/Fl*^ mice have been described in detail elsewhere [39]. Finally, the observed more aversive behavior of the mice is in line with the autistic features described in RSTS patients [40]. This further supports this new mouse model as a suitable model for studying basic pathophysiological processes that may lead to the development of intellectual disability and cognitive impairment in RSTS.

### The role of CBP in forebrain development

Though of high importance for forebrain and cognitive development - as seen from the example of RSTS - the early depletion of CBP in the used model does not halt forebrain development completely, and the general organization is conserved. Thus, although perturbed, the neocortex with its six layers, the hippocampus and the OBs are developed, and animals are viable. Nevertheless, the CBP depletion in the used model occurs after neural tube formation and onset of neurogenesis [51, 56]. Hence, it cannot be ruled out that CBP is indeed indispensable for general forebrain structuring or neural tube formation. Although general forebrain development proceeds, a loss of CBP leads to smaller brain, neocortex, hippocampus, OB and CC sizes in the analyzed knockout model, supporting previous postulations of its important role in brain size regulation. A heterozygous loss of CBP has been shown to lead to microcephaly, a smaller CC and a reduced OB size, both in mouse models as well as in RSTS patients [1, 3, 40].

### Developmental processes depending on CBP function

Previous studies suggested an essential role of CBP in neural differentiation [2, 65]. This is supported by the few existing neuropathological reports of autopsies of RSTS patients, which all describe altered cortical neuron morphology with cells appearing less differentiated and smaller [13, 18, 49]. An effect of CBP on neuron morphology has recently been demonstrated in a similar conditional mouse model using the *Nestin* promoter, which is in line with our observations [15]. Moreover, whereas incomplete siRNA knockdown and postnatal conditional CBP knockout in principal neurons did not show alterations in cell survival- and proliferation rates, early CBP depletion leads to increased apoptosis and less proliferation of NPCs within the germinal VZ during forebrain development [62, 65]. This suggests a higher dependence of NPCs on CBP compared to already senescent neurons. Accordingly, it has been shown before that CBP expression levels decrease during development [65]. In line with these findings, adult neurogenesis was also found to be impaired in *hGFAP-cre::CBP*^*Fl/Fl*^ mice, especially in the hippocampus, as reflected by fewer stem cells and transit amplifying cells.

Another important developmental process affected by the early loss of CBP was NPC migration. In *hGFAP-cre::CBP*^*Fl/Fl*^ mice NPC migration was delayed or even disturbed during forebrain development as well as postnatally. We propose that the migration deficit is mediated at least partially by IGF1. The phenotype of IGF knockout mice resembles aspects of our CBP knockout mice with smaller OBs and a cell accumulation at the V-SVZ [24]. It was shown that IGF1 is essential for the exit of neuroblasts from the RMS into the OB. This is in line with the results from our in vitro migration assays, where we could observe a reduced number of single cells leaving the V-SVZ explant in the presence of CBP deficient OB tissue and a rescue of this effect by IGF1.

### Implications for RSTS pathogenesis

Previously, neocortex and hippocampus have been postulated as main anatomical substrates of intellectual disability and cognitive impairment in RSTS [62, 65, 71].

It has been shown that a postnatally reduced KAT activity leads to deficits in hippocampal synaptic plasticity and memory storage [30, 64, 71]. Additionally, adult neurogenesis has been shown to be important in memory formation, a process, which is disturbed in our mouse model [25, 55]. We show that CBP plays a central role for the correct development of forebrain structures and for appropriate neural differentiation. Neocortex, hippocampus and CC development are considerably impaired after early loss of CBP leading to the conclusion that their function is disturbed as well.

It has been described extensively that intellectual disability syndromes such as Down-, Rett- and fragile X-syndrome are associated with an altered neuron and spine morphology [28]. The reported abnormalities in the dendritic spine length, branching and spine number are similar to the findings in *hGFAP-cre::CBP*^*Fl/Fl*^ mice suggesting a functional significance in intellectual disability. The reduced number of synapses in the CBP deficient animals supports the hypothesis that neurons are morphologically and functionally affected by a CBP deficiency.

It has been suggested that RSTS - so far attributed mainly as multiple congenital anomalies and intellectual disability syndrome - should be included in the family of neuronal migration disorders due to commonalities with holoprosencephalic and arhinencephalic conditions as observed through in vivo MRI based volumetry of CBP haploinsufficient mice [3]. The findings of reduced OB and neocortex sizes as well as the profoundly disturbed migration processes in *hGFAP-cre::CBP*^*Fl/Fl*^ mice and MR images of RSTS patients strongly support this assumption and make it very likely that disrupted migration processes during brain development play an important role in RSTS pathogenesis [1]. Nevertheless, these findings do not permit an assumption, which pathophysiological process predominantly causes the development of the typical RSTS related symptoms. It has been proposed lately, that the main function of KATs is the integration of environmental signals into gene expression and that this crosstalk is also responsible for the wide variety of symptom strength in RSTS patients [57]. This is further strengthened by the analysis of the time-resolved acetylome of CBP which revealed very rapid changes in acetylation making it even more plausible that acetylation is able to react to changes in the environment quickly [67]. Together, the development of RSTS should not be attributed to one disrupted process during development but is the result of continuous interference between multiple processes that are affected by diminished availability of CBP or its KAT activity.

## Materials and methods

### Gene mutation type & location in published cases of RSTS

To investigate the role of the KAT and other domains of the CBP gene for the development of RSTS, 193 published cases of RSTS listed in the Human Gene Mutation Database (Qiagen Bioinformatics) were grouped by type and localization of the pathogenic mutation as follows: 97 cases of RSTS caused by point mutations; 34 cases of RSTS caused by duplications, insertions & indels; 62 cases of RSTS caused by small deletions; 59 cases of RSTS caused by large deletions (not used); 54 cases of RSTS caused by missense mutations regardless the location within the CBP gene; 36 cases of RSTS with missense mutations within KAT domain; 9 cases of RSTS with missense mutations in exons 1–17 (before KAT domain), 43 cases of RSTS with nonsense mutations regardless the location; 16 cases of RSTS with nonsense mutations in the KAT domain, 21 cases of RSTS with nonsense mutations in exons 1–17 (before KAT domain). We focused on point mutations for a more precise allocation concerning different domains and exons. To compare the mutation distribution between different areas of the gene, the numbers of point mutations per 100 bp (base pairs) rates were calculated for exons, certain domains, or regions of interest to take the different region sizes into account. Cases, in which the pathogenic mutation did not lie within the *CBP* exons, were not included.

### Transgenic animals

The generation of both *hGFAP-cre* [8, 75] and *CBP*^*Fl/Fl*^ [73] transgenic mouse lines has been described previously. All animal procedures were performed in accordance with applicable animal protection laws. All experiments were approved by the state of Bavaria under license number 55.2–1-54-2532-10-14 and the state of Hamburg (Reference 113/16). Animal handling was done in accordance with local governmental and institutional animal care regulations.

### Animal treatments

For measuring the proliferation rate in vivo, 25 μg bromodeoxyuridin (BrdU) or 5-ethynyl-2′-deoxyuridine (EdU) per gram bodyweight were injected intraperitoneally 2 h before sacrificing the animal. If indicated, a BrdU/EdU double pulse fate-mapping method was utilized. The double pulse method consisted of two injection steps. First, BrdU was injected and after a chosen interval, EdU was applied and the animal was sacrificed. For electron microscopy an in vivo perfusion with 4% paraformaldehyd was used as described elsewhere [19].

### Behavior testing

Different behavior tests were conducted to characterize *hGFAP-cre::CBP*^*Fl/Fl*^ mice. All tests were conducted between 9 am and 9 pm. The animals were accustomed to the test room for at least 24 h prior to testing. Gender-matched litter mates were used as a control group. All tests were performed with animals at P30. The animals’ genotypes were unknown to the tester during testing. Females and males were tested separately but indiscriminately included in the analysis.

An anxiety/curiosity light/dark test was utilized as published with a standard box size (60 × 40 × 40 cm, length x width x height). Latency until the first transition from the dark to bright compartment, the number of transitions and the total time spent in each compartment were measured.

An open field test was performed to analyze anxiety. Mice were subjected to an empty cage and their behavior was videotaped for 2 min. Afterwards, the video was analyzed for total distance travelled, time spent in center and vertical activity. The mouse path was tracked manually.

To investigate, whether structural and histological findings in *hGFAP-cre::CBP*^*Fl/Fl*^ transgenic mice reflected disorders of functional systems, a modified buried food and an olfactory habituation/dishabituation test [35, 72] were used. In the buried food test (BFT), the time until the mice had dug up a buried piece of food, was measured. For habituation/dishabituation testing plastic cartridges carrying a piece of cotton impregnated with 20 μl of either almond or banana extract were presented to the mice repeatedly. For the first 6 trials, almond extract was used for examining habituation, and in a 7th trial, banana extract was used as a novel scent to trigger dishabituation.

### Genotyping

For genotyping, tail or ear biopsies were used. DNA was extracted by tissue lysis with Laird’s buffer (200 mM NaCl, 100 mM Tris-HCl pH 8.3, 5 mM EDTA, 0.2% SDS, 200 μg/ml protein kinase K in ddH_2_O) and Isopropanol precipitation. DNA was dissolved in TE buffer (20 mM Tris-HCL pH 8.3, 1 mM EDTA in ddH_2_O) and stored at 4 °C. Genotype-specific regions of the genome were amplified via PCR utilizing primers described in the original publications (Cre: TCCGGGCTGCCACGACCAA, GGCGCGGCAACACCATTTT, CBP: CCTCTGAAGGAGAAACAAGCA, ACCATCATTCATCAGTGGACT) and a TAQ-Polymerase (Promega) based standard reaction mixture.

### Mouse MRI data

All measurements were conducted in T2 weighted images on freshly sacrificed animals. At least 3 animals per genotype were used. Pictures were analyzed by manual quantification using MRIcro software (Chris Rorden, Version 1.40).

### Human MRI

The procedure and study design were approved by the ethics committee of the faculty of medicine of the Ludwig-Maximilians-University in Munich. In this retrospective analysis, images were analyzed in a pseudonymized manner. The children’s age at the time of MRI ranged from 1 month to 5 years. All measurements were performed on T2–weighted and T1-weighted sequences in axial, coronal and sagittal orientation. To perform matched-pair statistical analysis analogue measurements were conducted on cranial MR imaging data from patients with known RSTS and age-matched control subjects retrospectively identified in the institutional database.

### Histology and immunohistochemistry

After dissection of mice, the brain was prepared for staining procedures with standard procedures. Tissue that was meant to be analyzed through confocal microscopy for 3D cell reconstruction was fixated overnight in 4% paraformaldehyde in PBS at 4 °C and then processed to 100 μm slices using a VT1000S microtome (Leica Biosystems). Tissue destined for light or fluorescence microscopy was fixated in 4% formaldehyde solution, embedded in paraffin and 3 μm sections were cut. General morphology was analyzed by hematoxylin/eosin (H&E) staining, following a standard protocol.

For immunohistochemical procedures, standard procedures including dewaxing and rehydration, antigen retrieval, endogenous peroxidase inactivation, antigen blocking (I-Block casein-based blocking reagent (ThermoFisher Scientific)) and detection were performed. Primary antibodies used in this study were as follows: BrdU (Roche #11170376001, 1:500), BrdU clone Mobu-1 (Invitrogen #B35128, 1:100), Calbindin (Chemicon #AB1778, 1:100), Caspase 3 (Cell Signaling Tech #9664, 1:00), CBP (Biozol #LS-B3360, 1:50), Cre (Covance #PRB-106P, 1:3000), HuB (Sigma #H1538, 1:200), Ki67 (Abcam #ab16667, 1:200), MBP (Abcam #ab40390, 1:100), NeuN (Abcam #ab104224, 1:300), Pax6 (DSHB #Pax6, 1:25), Prox1 (Abcam #ab199359, 1:500), Sox2 (Abcam #ab79351, 1:200), Tbr2 (Millipore #AB2283,1:300), Wfs1 (Proteintech #11558–1-AP, 1:50). Detection was achieved by using the DAKO EnVision™Plus System, HRP following the manufacturer’s instruction or for immunofluorescence staining, with species-specific fluorophore linked secondary antibodies (Alexa 546 Invitrogen **#** A-11003 and DAPI Roth #28718–90-3).). EdU positive cells were stained by using the Click-IT® assay (ThermoFisher #C10637). The 100 μm slices for 3D cell reconstruction were stained directly with NeuroTrace 530/615 (1:100; ThermoFisher #N21482), and Hoechst (1:1000; Invitrogen #H3570) as a nuclear counter staining. VECTASHIELD HardSet Antifade mounting medium (VECTOR Laboratories) was used for mounting.

### Stereological measurements

To increase validity and reduce variability a stereological approach was chosen for analyzing the different forebrain structures. For each structure of interest three section planes were analyzed in every animal of the *hGFAP-cre::CBP*^*Fl/Fl*^- or control group. In each section plane, several measurements were executed and averaged for parameters. Section planes were chosen by recognizable landmarks in light microscopy instead of predetermined intervals as brain size was not a stable constant. All measurements were conducted on pseudorandomized images with the help of the open source image processing program ImageJ.

### 3D cell volume reconstruction of layer V giant pyramidal cells

For 3D cell reconstruction z-stack series in layer V of the neocortex were acquired from 100 μm slices stained with NeuroTrace & Hoechst using a Zeiss LSM780 confocal microscope and ZEN microscope software (Zeiss). The z-stacks acquired through confocal microscopy were further analyzed through custom-written Matlab analysis using the microscopy image analysis software Imaris (Bitplane) to result in 3D reconstructions of neual cell somata and cell cores.

### Golgi-Cox staining and quantification

Golgi-Cox staining of adult mouse brains was performed using the FD Rapid GolgiStain Kit (FD NeuroTechnologies, INC.) according to the instructions from the manufacturer. Briefly, freshly dissected brains were impregnated with a premade solution of mercuric chloride, potassium dichromate and potassium chromate for 1 week, cut in 200 μm slices with a vibratome (Leica) and the impregnation was visualized with a solution provided by the manufacturer. For quantification of dendrite length, numbers of branches per dendrite and spine density, 10 representative pictures per mouse were taken and three mice per genotype were used. For analysis of spine types, 10 neurons per mouse were analyzed.

### Electron microscopy

Electron microscopy was performed on adult mice perfused with 4% PFA. The brain was dissected immediately after perfusion and stored in 2% PFA + 2% Glutaraldehyde. A piece of cerebral cortex was cut from a frontal slice of the brain and prepared for EM. Samples were washed in 0.1 M cacodylate buffer (Sigma-Aldrich), incubated for 2 h in 1% osmium tetroxide (Science Services, Munich, Germany), dehydrated in an ascending series of ethanol, and embedded in Epon 812 (Serva). Ultrathin sections were counterstained with uranyl acetate (Polyscience, Eppelheim, Germany) and lead citrate (Riedel-de Haën, Seelze, Germany), and analyzed with a LEO 912 AB OMEGA electron microscope (Leo Elektronenmikroskopie, Oberkochen, Germany).

### SVZ and OB explant culture

Cell explants of SVZ were prepared as previously described in [68]. Briefly, brains of 2 to 5 day old mice were dissected, freshly cut with a vibratome (Leica) and the SVZ dissected. The SVZ was cut and put in 50 μl Matrigel (Corning). The explants were cultured in 500 μl culture medium (Neurobasal (Gibson), B27, 0.5 mM L-Glutamine, 50 U/ml Pen/Strep) in a 24 well plate. OB explants were kept in the same medium in 30 μl Matrigel. Cultures were grown for 48 h in a humidified incubator at 37 °C and 5% CO_2_. In case of medium exchange cultures, the medium was changed after 24 h. After 48 h, pictures of the cultures were taken and migration distance or single cell number was determined. Per experiment, at least 6 explants per condition were analyzed and three independent experiments were conducted. If indicated, 200 ng recombinant IGF1 (PeproTech) were added to the culture medium.

### RNA sequencing

After isolation of total RNA the RNA integrity was analyzed with the RNA 6000 Nano Chip on an Agilent 2100 Bioanalyzer (Agilent Technologies). From total RNA, mRNA was extracted using the NEBNext Poly(A) mRNA Magnetic Isolation module (New England Biolabs) and RNA-Seq libraries were generated using the NEXTFLEX Rapid Directional qRNA-Seq Kit (Bioo Scientific) as per the manufacturer’s recommendations. Concentrations of all samples were measured with a Qubit 2.0 Fluorometer (Thermo Fisher Scientific) and fragment lengths distribution of the final libraries was analyzed with the DNA High Sensitivity Chip on an Agilent 2100 Bioanalyzer (Agilent Technologies). All samples were normalized to 2 nM and pooled equimolar. The library pool was sequenced on the NextSeq500 (Illumina) with 1 × 75 bp, with 16.1 to 18.6 mio reads per sample.

For each sample the sufficient quality of the raw reads was confirmed by *FastQC* v0.11.8 [54]. Afterwards, the reads were aligned to the mouse reference genome GRCm38 with *STAR* v2.6.1c [16] and simultaneously counted per gene by employing the *--quantmode GeneCounts* option. Counts are based on the Ensembl annotation release 95. Differential expressed genes were estimated with *DESeq2* v1.22.2 [37].

### Statistical analysis

Statistical analysis was conducted with Prism (Versions 5.0, 6.0 and 7.0) software (GraphPad). If not stated otherwise, all data presented are mean ± s.e.m., with *n* = 3 for each group and each data point represents an individual animal or an independent experiment. *P* values < 0.05 were considered significant (*p < 0.05, **p < 0.01, ***p < 0.001, ****p < 0.0001). By default, the unpaired *t* test (two-tailed) was applied to compare the means of two groups if not stated otherwise in the figure legend. The Χ^2^ (Chi-squared) test was used for comparing point mutation frequencies of specific domains and regions of the CBP gene. Further, a two-way repeated measurements ANOVA test and Bonferroni’s multiple comparisons post hoc test as well as nonlinear regression using an exponential decay model were applied for evaluation of the habituation test.

## Supplementary information

**Additional file 1: Figure S1.** Mutation distribution analysis in published RSTS cases and additional MRI results. **Figure S2**. Immunohistological analysis of the hippocampus, neocortex and olfactory bulb, myelination analysis and olfactory behavior testing. **Figure S3**. Immunohistological analysis of prenatal stages E14.5 and E16. **Figure S4**. Cell volume and dendrite branch analysis. **Figure S5.** Supplementary data on neurogenesis and migration in the olfactory bulb, the V-SVZ and the corpus callosum at P15 and P30.

## Data Availability

The data that support the findings of this study are available from the corresponding authors, [M.S., M.L. & U.S.], upon request.
