# Supplementary material for: The transcriptional coactivator and histone acetyltransferase CBP regulates neural precursor cell development and migration
Source: Acta Neuropathol Commun. 2019 Dec 5;7:199. doi: 10.1186/s40478-019-0849-5 (PMC6896766; doi:10.1186/s40478-019-0849-5)
Supplement: Supplementary file 1 — Additional file 1: Figure S1. Mutation distribution analysis in published RSTS cases and additional MRI results. Figure S2. Immunohistological analysis of the hippocampus, neocortex and olfactory bulb, myelination analysis and olfactory behavior testing. Figure S3. Immunohistological analysis of prenatal stages E14.5 and E16. Figure S4. Cell volume and dendrite branch analysis. Figure S5. Supplementary data on neurogenesis and migration in the olfactory bulb, the V-SVZ and the corpus callosum at P15 and P30. [file 40478_2019_849_MOESM1_ESM.pdf]

Supplement Figure 1

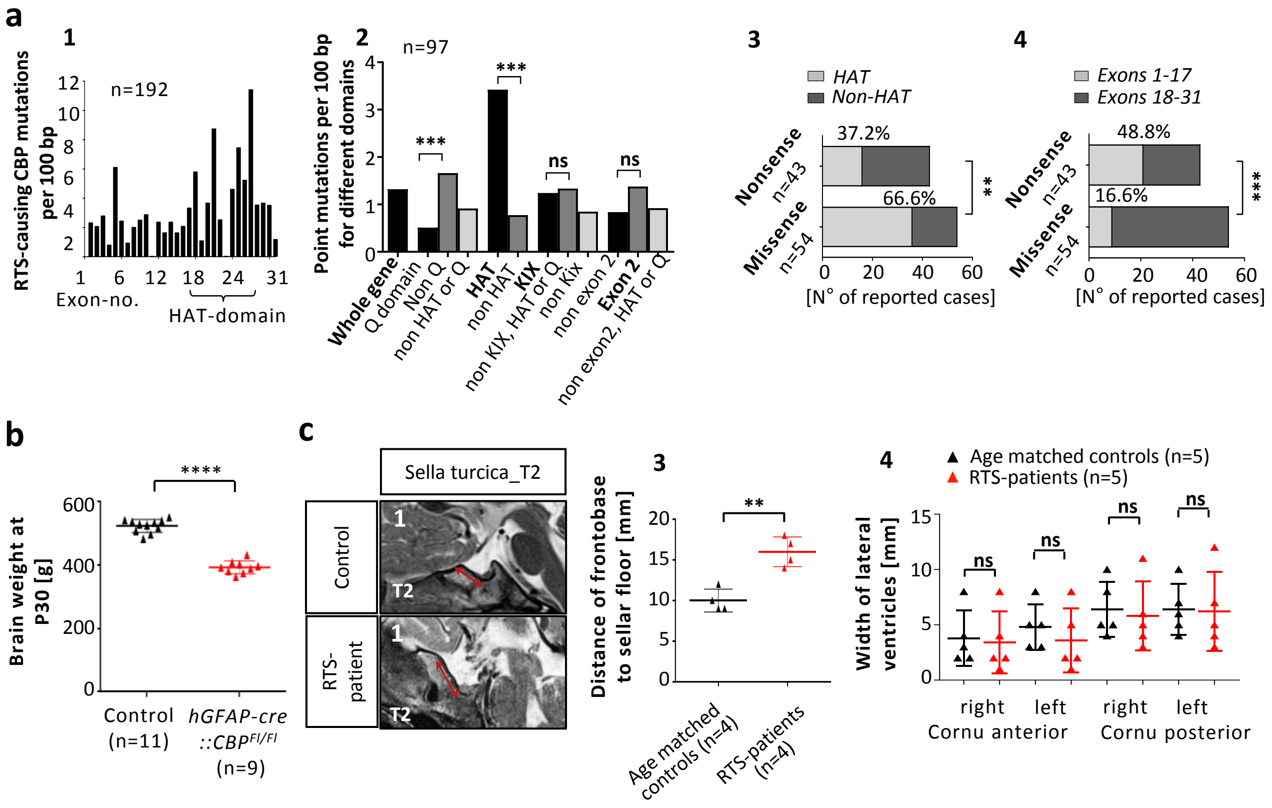

Fig. S1: (a1) Published RSTS causing point mutations, duplications, insertions, indels and small deletions as mutations per 100 bp sorted by affected exons with a visible peak of mutations within the KAT domain (Exons 18-29; 52/192 published RSTS cases) **(a2)** Point mutations per 100 bp distribution analysis comparing different domains and regions of interest. KAT domain harbors significantly more RSTS causing point mutations, the C-terminal Q domain significantly less. **(a3-4)** Comparison of nonsense and missense mutation counts in the KAT domain and in exons 1-17, both as percentage of all reported cases of RSTS-causing point mutations. A significantly bigger percentage of all reported missense mutations compared to the percentage of all nonsense mutations (66.6% to 37.2%) were found within the KAT domain. The percentage of all reported nonsense mutations in exons 1-17 were found to be significantly higher than the respective percentage of missense mutations (48.8% to 16.6%) **(b)** Brain weight was significantly lower in *hGFAP-cre::CBP*$^{Fl/Fl}$ transgenic animals at P30. **(c1-3)** Significantly increased distance between the base of the frontal lobe and sellar floor of RSTS patients in sagittal T2-weighted MR sections marked with red arrows. **(c4)** Width measurements of the lateral ventricle horns without significant differences between RSTS patients and age-matched control children. *$**p < 0.01$, $***p < 0.001$*

Supplement Figure 2

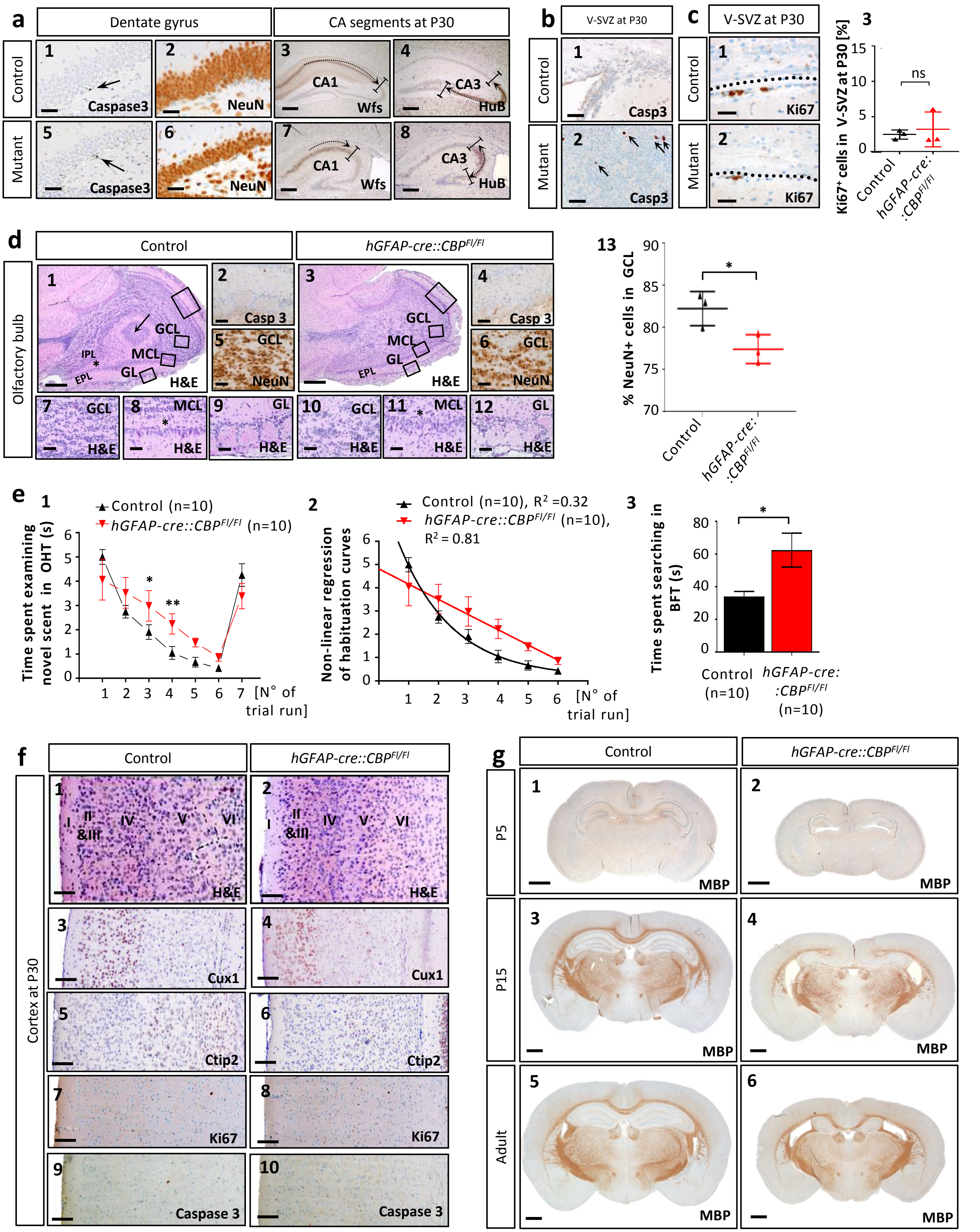

Fig. S2: (a1,5) Caspase 3 stained sections of the dentate gyrus at P30. **(a2,6)** NeuN staining shows an unaltered expression of the neural marker NeuN in the granular cell layer. **(a3,4,7,8)** Wfs1 CA1 segment staining & HuB CA3 segment staining. Compared to control animals, no difference in hippocampus segmentation was found in *hGFAP-cre::CBP*$^{Fl/Fl}$ animals. **(b1-2)** Caspase 3 staining shows significant apoptosis within the cell accumulation as marked by arrows. **(c1-3)** Ki67 staining of frontal sections of the V-SVZ at P30. No statistical significant difference in proliferation rates in the V-SVZ at P30. **(d1,3,7-12)** Sagittal H&E stained sections of the OB with GCL, internal plexiform layer (IPL,*) mitral cell layer (MCL), external plexiform layer (EPL) and glomerular layer (GL). The arrow marks the continuation of the rostral migratory stream (RMS) within the olfactory bulb. This structure as well as the IPL (indicated by an asterisk) were not consistently determinable in *hGFAP-cre::CBP*$^{Fl/Fl}$ mice. Boxes mark the areas used for Caspase 3 apoptosis analysis. **(d2,4)** No relevant amount of apoptotic Caspase 3+ cells were observed in the OB of *hGFAP-cre::CBP*$^{Fl/Fl}$ or control animals. **(d5,6)** NeuN staining of the GCL. **(d 13)** Rate of NeuN positive cells in the GCL, significantly lower after early loss of CBP. **(e1)** Habituation/ dishabituation test: transgenic mice spent significantly more time examining the cartridge carrying the novel scent when presented for the 3rd and 4th time. **(e2)** Non-linear regression of habituation curves. A one phase exponential decay model (Y=(Y0 - Plateau)K*X + Plateau) was used for non-linear regression. The *hGFAP-cre::CBP*$^{Fl/Fl}$ could not be fitted in an exponential manner (R2=0.32), whereas regression of the control curve led to a rather good fit (R2=0.81). **(e3)** Buried food test (BFT): Mutant animals spent significantly more time searching. **(f1-6)** Frontal sections of the neocortex show the establishment of six neocortical layers after CBP depletion (I-VI). Cux1 was used to specifically stain layers II-IV and Ctip2 for layers V and VI. **(f7-10)** No relevant amount of proliferation or apoptosis was observed in frontal Ki67 and Caspase 3 stained sections of the neocortex at P30. **(g1-6)** MBP (myelin basic protein) myelination staining of frontal sections at P5, P15 and P30. *Scale bar: 40 µm (a1,2,5,6, b1,2)***,** *200 µm (a3,4,7,8) 30 µm (c1-2), 220 µm (d1,3), 130 µm (d2,4), 70 µm (d5-12), 200 µm (f1-10), 1250 µm (g1,2), 1000 µm (g4-6); *p < 0.05, **p < 0.01*

Supplement Figure 3

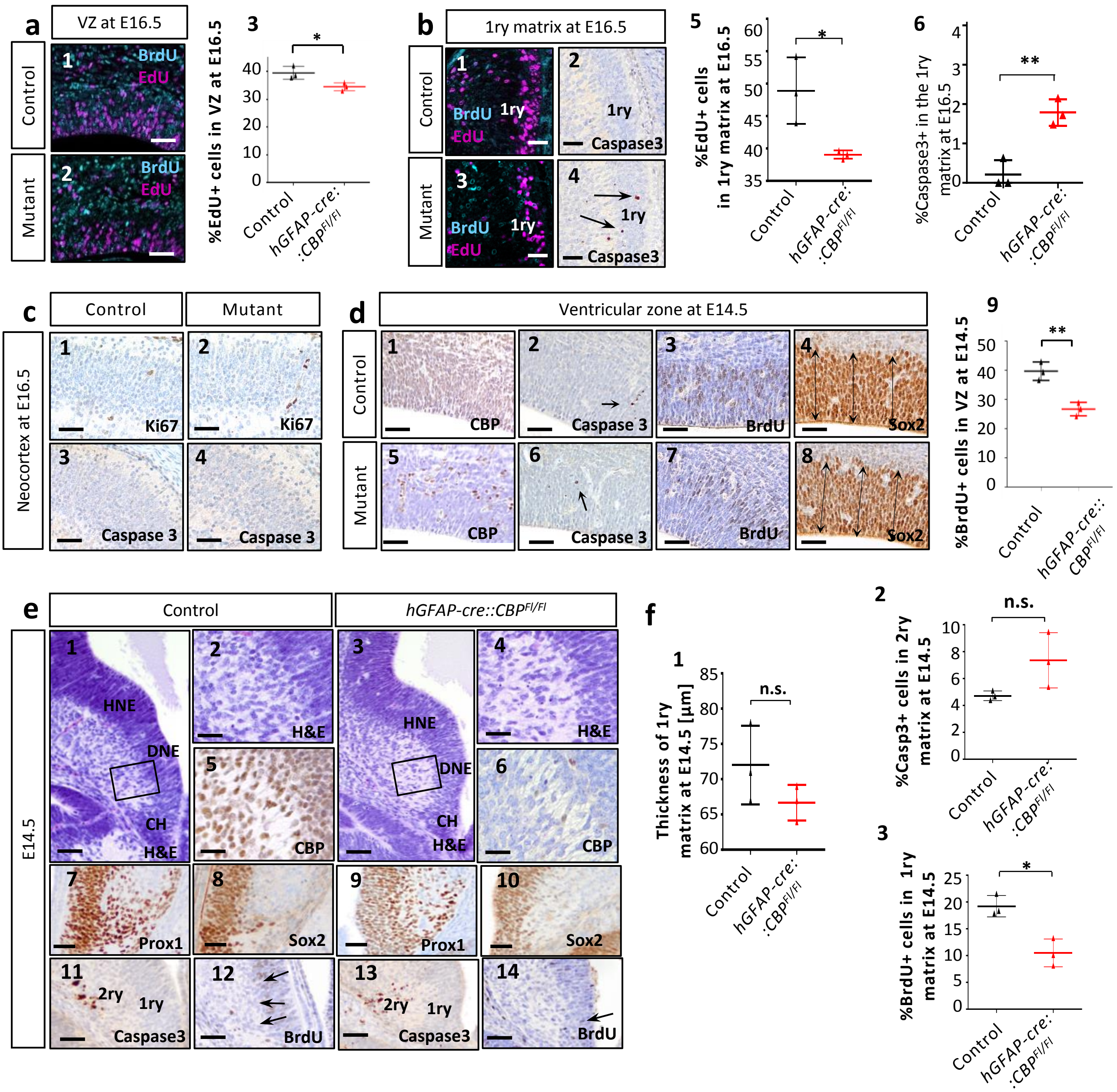

Fig. S3: (a1,2) Frontal sections of the VZ at E16.5 with EdU-BrdU co-staining (BrdU injection at E14.5, EdU injection at E16.5). **(a3)** A significantly reduced proliferation rate was measured within the ventricular zone of transgenic mice. **(b1-4)** Frontal sections of the DNE at E16.5 with EdU-BrdU co-staining for proliferation analysis and fate mapping and Caspase3 apoptosis staining (BrdU injection at E14.5, EdU injection at E16.5). **(b5,6)** Significant increase of apoptosis and a significant decrease of proliferation in the DNE at E16.5 after early CBP depletion. **(c1-4)** Frontal sections of the forming neocortex at E16.5 with Ki67 proliferation and Caspase3 apoptosis staining. No significant amount of proliferating or apoptotic cells was observed. **(d1-8)** Confirmation of extensive CBP knockout in transgene animals, Sox2 stem cell staining, BrdU proliferation staining and Caspase3 apoptosis staining in high-power magnifications of the ventricular zone at E14.5 (BrdU injection 2 h before sacrifice). **(d9)** A significantly reduced BrdU+ rate and thus proliferation rate was found in the ventricular zone at E14.5. **(e1,3)** Overview over hippocampal formation at E14.5 in H&E staining of frontal sections. Hippocampal neuroepithelium (HNE), dentate neuroepithelium (DNE) and cortical hem (CH). **(e2,4-6)** Dentate neuroepithelium and ongoing cell migration with complete CBP knockout in transgenic animals at E14.5. **(e7,9)** Prox1 staining at E14.5 confirming dentate granule neuron lineage character of migrating cells in both control and transgenic mice. **(e8,10)** Sox2 staining showing stem cell character in the dentate neuroepithelium at E14.5. **(e11-14)** 1ry and 2ry matrix in Caspase 3 and BrdU stained frontal sections at E14.5 **(f1)** Thickness measurements of the 1ry matrix (DNE) in Sox2 stained frontal sections at E14.5, no significant differences between control group and transgenic animals. **(f2)** No significant difference in the apoptosis rate among the migrating cells within the 2ry matrix was measured between the two groups. **(f3)** BrdU+ and thus proliferation rate in the 1ry matrix, significantly reduced in *hGFAP-cre::CBP*$^{Fl/Fl}$ mice at E14.5.

*Scale bar: 50 µm (a1,2; c1-4; d1-8), 30 µm (b1-4), 35 µm (e1,3), 15 µm (e2,4-6), 25 µm (e7-14); *p < 0.05, **p < 0.01.*

Supplement Figure 4

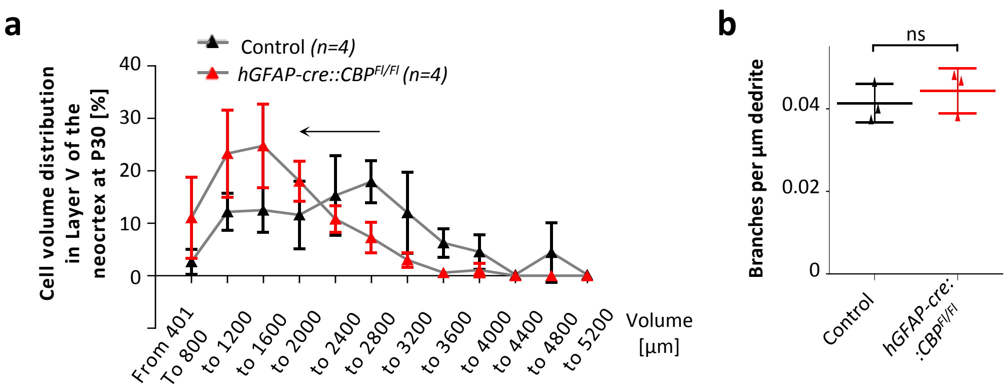

Fig. S4: (a) Cell volume distribution curves with rates of cells within a certain volume range. Error bars indicate inter-individual differences. The curve for neurons from the *hGFAP-cre::CBP*$^{Fl/Fl}$ group is shifted to the left towards smaller cell volumes as indicated by the arrow. **(b)** The number of branches per µm apical dendrite is unchanged in *hGFAP-cre::CBP*$^{Fl/Fl}$ mice.

Supplement Figure 5

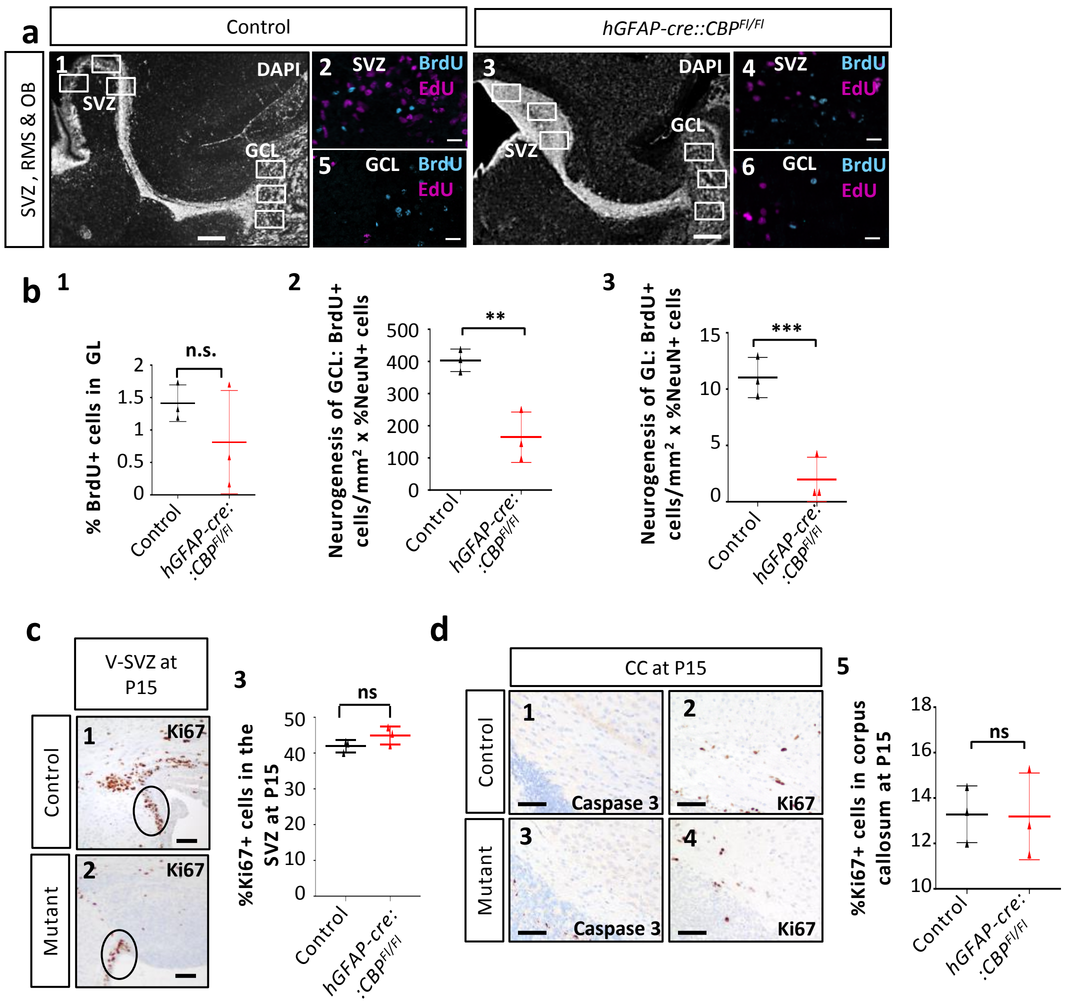

Fig. S5: (a1,3) Overview of the ventricular-subventricular zone (V-SVZ), rostral migratory stream (RMS) and granular cell layer (GCL) of the OB in sagittal sections at P30 (DAPI). Boxes mark areas, in which migration analysis was conducted **(a2,4-6)** In BrdU/EdU immunofluorescence staining of the V-SVZ and GCL no cells that were both BrdU+ and EdU+ were found. **(b1)** BrdU+ rate in the GL, no significant difference between the two groups. **(b2,3)** Neurogensis estimated in the granular cell layer and the glomerular cell layer of the olfactory Bulb as BrdU+ cells per mm^2 times the NeuN+ rate in the respective area. Neurogenesis was shown to be significantly reduced in both the granular cell layer and the glomerular layer of *hGFAP-cre::CBP*$^{Fl/Fl}$ mice. **(c1-3)** Ki67-staining of the V-SVZ at P15. No significant difference in proliferation rates. **(d1-4)** CC with apoptosis and proliferation staining in sagittal sections at P15. No increase of apoptosis in *hGFAP-cre::CBP*$^{Fl/Fl}$ mice. **(d5)** No significant difference was measured for the Ki67+ rate in the CC at P15 between transgenic and control animals. *Scale bar: 250 µm (a1,3), 30 µm (a2,4-6), 75 µm (c1-2), 200 µm (d1-4);* $^{**}p < 0.01$, $^{***}p < 0.001$
